# Web-based self-management for young cancer survivors: consideration of user requirements and barriers to implementation

**DOI:** 10.1007/s11764-014-0400-4

**Published:** 2014-09-19

**Authors:** Louise Moody, Andrew Turner, Jane Osmond, Louise Hooker, Joanna Kosmala-Anderson, Lynn Batehup

**Affiliations:** 1 Department of Industrial Design, Coventry School of Art and Design, Coventry University, Priory Street, Coventry, CV1 5FB UK; 2 Applied Research Centre in Health & Lifestyle Interventions, Faculty of Health and Life Sciences, Coventry University, Priory Street, Coventry, CV1 5FB UK; 3 Teenage and Young Adult Cancer Service, University Hospital Southampton, Tremona Road, Southampton, SO16 6YD UK; 4 Macmillan Cancer Support, 89, Albert Embankment, London, SE1 7UQ UK

**Keywords:** Web-based self-management, Teenage and young adult, Cancer survivors, Online support, E-learning

## Abstract

**Purpose:**

As the population of young cancer survivors increases, there is a need to develop alternative ways of providing post-treatment support. Online systems potentially offer self-management and e-learning support following cancer treatment. This research aims to explore the self-management support needs of teenage and young adult cancer survivors and consider whether those needs can be met through a web-based self-management resource.

**Methods:**

A mixed methods approach was adopted including an online survey (*n* = 24), focus groups and interviews with teenage and young adult cancer survivors (*n* = 7) and interviews with parents of survivors (*n* = 6), information technology specialists (*n* = 8) and clinical, nursing and social work professionals (*n* = 11).

**Results:**

All stakeholders were supportive of web-based self-management to meet information and support needs that would supplement continued direct interaction with clinical staff. Barriers to implementation were identified in terms of risks to young people, governance issues and the challenges of providing a long-term service.

**Conclusion:**

Computer access and use amongst teenagers and young adults is commonplace, and there is an expectation that self-management needs will be met at least partially online in the future. There is a desire for online social support through peer interaction as well personal developmental and clinical management. These elements may need to be run through different systems to cater for governance requirements.

**Implications for Cancer Survivors:**

An online self-management system could provide support at a number of different levels. The barriers to implementation should be addressed, to ensure that survivors can be supported in this way in the future.

## Introduction

Cancer survivors aged between 16 and 24 years have distinct needs [1, 2]. The transition from older adolescence to young adulthood presents a wide range of educational, behavioural, physical and psychosocial challenges and needs for teenage and young adult (TYA) cancer survivors [2–6]. During this period, young people typically develop a positive body image, establish an identity, separate from parents, begin dating and start to make decisions about careers and family [7]. When recovering from cancer, young people face additional and distinct challenges including premature confrontation with mortality, changes in physical appearance, increased dependency on parents, disruptions to social life and school/employment, loss of reproductive capacity and health-related concerns about the future [7, 8]. As the survivor population grows, it is increasingly important to provide ongoing emotional and practical support for the adjustment to the effects of cancer as well as addressing ongoing self-management issues [9]. The care offered should take into account specific developmental characteristics [10].

Studies have shown a need for age-appropriate information and education about cancer, fertility, reproductive risks and lifestyle; complementary and alternative health services; relaxation techniques and stress management; mental health counselling; and retreat programmes [3, 5, 10]. Individualised information may be required on the risk of recurrence, monitoring for long-term treatment effects, support during the transition from oncology to a primary care setting and evidence-based guidelines for long-term follow-up care [3]. Young adult cancer survivors themselves give high priority to meeting other cancer survivors to gain support and help with coping with and expressing cancer-related emotions and feelings [3, 5].

Despite these wide-ranging requirements, educational and support programmes for young adult cancer survivors are scarce, and the needs are not always met [10]. In the USA, only 53 % of young cancer survivors were found to receive long-term follow-up care, with only 8 % receiving care with involvement of a primary care physician [11]. Evaluation studies looking at uptake and effectiveness for support through social networking [12], video sharing [12], retreats [13], workshops [14] and group events [15] have shown that regardless of delivery mode, participants on all programmes found the opportunity to share their feelings and experiences with others helpful and satisfying. Programmes that included an educational component seemed to enhance participants’ knowledge of their condition and potential late treatment effects [12–16]. However, key system-driven barriers include insufficient resources and finances to develop and sustain programmes, low institutional commitment to provide survivorship care and poor communication with community physicians [17]. Patient-driven barriers include lack of interest in, and awareness of existing programmes [17], low confidence in managing survivorship care and psychosocial and physical barriers [5].

It is argued that the barriers to some of the existing face to face programmes might be countered through the use of the internet. The internet and growth in social networking sites potentially offer a strong sense of community, a place for sharing experiences of cancer [14, 16, 18] and a platform for self-management and e-learning support [19]. With prolific use of the internet, it is perhaps surprising that there are still a limited number of evaluated web-based interventions; however, evidence does suggest that web-based interventions in other areas can have a positive impact on quality of life and other health, self-management and behavioural outcomes [18, 20–24]. A web-based resource for TYA therefore may help young survivors take greater responsibility for self-managing their recovery, guide them towards reliable and trustworthy information, and allow them to connect with one another from home [25].

The existence of several online systems for young people in the UK indicates the growing appetite for web-based support and management, with examples providing cancer survivor care plans, access to appropriate sources of advice and support and chat rooms [26–29]. Patients, parents and clinicians have been found to be supportive of increasing patient autonomy via online resources [20], with 88 % feeling comfortable with internet follow-up being delivered some or all of the time. Despite this, there are few systems in use that have been set up specifically for teenage and young adult cancer survivors with a recognised self-management approach to improving quality of life and psychosocial well-being. With this in mind, this research seeks to explore the self-management information and support needs of teenage and young adult cancer survivors.

## Aims

### This study aims to explore the following:

- The self-management support needs of teenage and young adult cancer survivors
- The desirability of online self-management with a range of stakeholders
- Barriers to online self-management systems for young adult cancer survivors

## Methods and materials

A mixed methods exploratory approach was adopted including the following:

- Focus groups and interviews with teenage and young adult cancer survivors and their parents
- An online survey with teenage and young adult cancer survivors
- Interviews with professionals including medical, nursing, social and youth, and information technology (IT) specialists

The study was approved by the Coventry University Ethics committee. All participants gave their informed consent prior to participation in the study.

### Focus groups and interviews with survivors and their parents

To ascertain the information and support needs of teenage and young adult cancer survivors in relation to self-management, teenage and young adult cancer survivors who received their treatment and aftercare at University Hospital Southampton NHS Foundation Trust (UHS) UK, and their parents were asked to participate in a focus group. Information letters were sent out from UHS staff by post to the 80 patients (aged 16–25 years) known at that time to the Southampton TYA support service regardless of the type of cancer they had received treatment for. The inclusion criteria dictated that participants were post-treatment, with up to 2 years since completing treatment, had received treatment as teenagers or young adults and were over 16 years old at the time of recruitment to the study. A letter was also sent inviting their parents to the parallel focus group. Those who expressed an interest in taking part contacted the CLIC Sargent^1^ Young People’s Community Worker for further details and to consent for their contact details being shared with the research team.

The focus groups (FGs) took place at St Mary’s Stadium, home of Southampton Football Club. Budget and time constraints meant that a pragmatic approach to collecting qualitative data was adopted involving a small number of respondents. The TYA cancer survivors received a £20.00 gift voucher for taking part.

Seven TYA participants (four females and three males) with ages ranging from 16 to 24 took part in a focus group and two telephone interviews. The two interviews were undertaken for participants willing to take part but unable to attend the scheduled focus group. A FG with six parents was conducted at the same time, in an adjacent room and comprised both parents/step parents of two of the teenage and young adult cancer survivors, and the mother of one, and the father of another survivor participant.

The semi-structured focus group and interview schedules were developed through consultation with the project steering group to ascertain the information and support needs of survivors in relation to self-management. Examples of questions asked of the TYA survivors included the following: What type of information was and support did you receive when you were diagnosed? What type of information did you want at the end of your treatment? Examples of questions asked of the parents included the following: What type of support did you receive when your child was diagnosed? What type of additional support did would you have found useful when your child was diagnosed? Drawn from the literature review, a number of potential features of a web-based self-management system were presented to potential users in the online survey (see Table 1) and through the focus groups and interviews.

**Table 1:** Potential features of a self-management system

• Self-management information (e.g. written materials, webinars, video podcasts)
• Informational resources /documents (e.g. long-term effects of treatment, what do my test results mean?)
• Case studies
• News section
• Ask a doctor/nurse (e.g. secure messaging to clinicians/live Q&A/Twitter chat hour/web chat live to doctors)
• Individual care pathways
• Making and cancelling appointments online
• Appointment reminders (e.g. text messaging/SMS or web-based)
• Looking up results
• Patient to patient communication (e.g. messaging between patients or parents, discussion forum)
• Parent information and access
• IT help link

The interviews lasted an average of 30 min (range 20–40 min). The focus groups lasted for 2 hour. They were conducted by two senior researchers from Coventry University who were unknown to the participants and are highly experienced health psychology and self-management researchers who have conducted many interviews with people affected by health conditions.

### Online survey with survivors

The design of an online survey was informed by findings from a review of literature describing existing support programmes for young cancer survivors and examining their needs and expectations regarding post-treatment support interventions, as well as the findings from the focus groups and interviews. Additional questions were added after consultation with members of the project steering group. We were unable to find an existing suitable validated measure.

The survey comprised of 47 closed questions. It asked open and multiple-choice questions about the usefulness of different features of online resources, preferred design features and potential barriers to using online resources. Examples of questions where respondents selected from a menu of options included the following: Having finished treatment, what did you find difficult to cope with? After finishing treatment, what information did you want? What features would you want to use on a teenage and young persons’ cancer website?

The survey was piloted with five young adult cancer survivors recruited who had attended (or who had expressed an interest but been unable to attend) the focus group. They completed the survey in full and were asked to provide feedback on how user friendly the questionnaire was, whether they understood the questions and whether they felt it covered all areas that it should. All pilot participants indicated it was easy to read, straightforward to complete and easy to follow. No one indicated any key issues missing. It took between 5 and 30 min to complete.

Following piloting, an invitation to participate in the survey was posted on the closed TYA Facebook page for Southampton TYA support service. An invitation was also texted or emailed to the other patients known to the Southampton TYA service (as per the list of 80 mentioned above) who were not members of the Facebook group and had not taken part in the focus groups. Having read the participant information sheet and indicating their consent, the participants were provided with the survey questions.

From the sample of 80 who received the invitation, 30 responses were received, of which 24 were fully completed surveys and included in the analysis. Respondents’ mean age was 21 (range 17–26). Participants had received treatment for Hodgkin’s lymphoma (*n* = 7), brain tumour (*n* = 6), leukaemia (*n* = 5), osteosarcoma (*n* = 4) and thyroid cancer (*n* = 2). Two respondents were still receiving treatment for the cancer. Respondents who completed their treatment attended hospital follow-up appointments every 3–6 months. The patients who were invited to complete the survey were over 16 years at time of study, so teenagers aged 13–15 at time of survey are not represented in these results. The cancers represented in this list are amongst those most prevalent on the TYA population. Other cancers prevalent in this age group, but not represented in the survey respondents, are testicular cancer, Ewing’s sarcoma and melanoma.

### Interviews with professionals

A list of health care and IT professionals at UHS as well as experts in the area of online cancer care were identified by the project steering group. A key factor for inclusion was availability for interview, as getting busy clinical staff for interview was an issue, and therefore there was an element of opportunity involved. All participants invited to interview agreed.

Nineteen semi-structured interviews related to online systems were conducted with a range of professionals. The sample included health care professionals (medical, nursing, social and youth workers) linked to UHS and working with teenage and young adult cancer survivors, IT-related participants working with NHS-based information technology, and those with experience of other online web-based support programmes for teenage and young adult cancer survivors. Recruitment continued until data saturation was reached—this was the point at which participants gave no new data. The interviews were undertaken by three researchers experienced in capturing requirements for system development from Coventry University. They were unfamiliar to the participants. Interviews were either undertaken face to face at UHW or on the telephone to suit the requirements of the participants.

The semi-structured interview schedule was developed through consultation with the project steering group to gain additional information to inform teenage and young adult cancer survivors support needs. It asked health care professional what kind of support in their experience young people cancer survivors need the most, what sources of support and information young people are using, and in their opinion how useful they are. They were also asked about the best format to provide information, the support and potential barriers for using those resources by young cancer survivors, the potential of the internet to support self-management and the potential risk of using this. IT professionals were asked specifically about IT requirements for the delivery, development and maintenance of a web-based, self-management intervention and establish potential barriers to success. Interviews were conducted face to face and over the telephone and lasted, on average, 30 min (range 20–40 min).

### Analysis

The interviews and focus groups were recorded, transcribed verbatim and analysed independently by two researchers using thematic analysis [30]. The online survey responses were tabulated and summarised graphically. More advanced statistical analysis was not appropriate given the sample size and explorative nature of the survey.

## Results

The findings have been combined to consider post-treatment self-management support needs and the desirability of an online resource across methods and participants.

### The need for support

Respondents across research methods felt that many teenage and young adult cancer survivors require ongoing emotional and psychological support well beyond the end of treatment, including emotional and practical support with getting back to normal life:

The emotional support that they need around that end of treatment, I would say is as big almost as at start of treatment (Health Services Professional/HSP).

Someone to talk to, I don’t have anyone to talk to anymore. ..... I mean there was so many questions that I want to ask, what’s going to happen to me when I grow up… (TYA)

Some young people (from the survey and focus groups) reported feeling isolated and alone after their treatment had ended. They still attended routine check-ups and appointments but they did not feel like they had time to ask questions or that consultants listened to their questions:

It just feels like I’ve been forgotten about, you’ve finished treatment, that’s it (TYA).

No, I think the same really, ..... forgotten about really. (TYA)

According to health care professionals interviewed, teenage and young adult cancer survivors experience a lot of difficulties when their treatment ends. They were aware that many feel down and unhappy, and feel like they have lost their main focus and support system. TYA reported feeling abandoned and left alone with their feelings and anxiety.

All of a sudden they’re not in hospital and feeling like they’re protected all the time, they’re back at home, and that’s when it starts to hit because your mind has got the time to start thinking oh my goodness I had cancer and I could have died, and then the worries about relapse and things (HSP).

Young people interviewed identified concerns about their personal support networks. They described losing friends who did not want to hear about their cancer experiences, or in some cases they felt judged by friends for having cancer. They also did not want to further burden their family:

Cancer, they thought it was a disease and you could get it. And literally, and then all the bullies were starting to be nice to me and it was just like go away, I don’t want you, but I lost all my friends. (TYA)

The interviews with health care professionals indicated that post-treatment support was currently provided from three main sources: the hospital (e.g. social workers, specialist nurses), online forums and social networks (e.g. chat forums, *Jimmy Teens TV*), and family. Across the participant groups, it was recognised that talking to others who had been through a similar cancer experience either through face-to-face or online contact was considered a very useful source of support:

They [young people] gain support from meeting other people who are on a similar journey as them and lots of them have become sort of lifelong buddies with people that are going through the same thing (HSP).

Some of the young survivors indicated they accessed cancer websites and made use of Facebook groups, and did so to access social support rather than seek clinical information. The importance of having the opportunity to share online or face-to-face their feelings and experiences with age-group peers who are going through similar experiences was emphasised:

You’re not glad that they’ve got cancer, but it’s nice to have someone to speak to about it. (TYA)

### The type of information and support needed

After completing treatment, young survivors in the survey sample indicated that they wanted access to *information* on long-term treatment effects (83 %), side effects (75 %) and fertility (58 %). Support needs were also explored. Fertility worries were identified as one of the most difficult issues to deal with after completing treatment. Half of all respondents (50 %) stated that they would like to receive *support* in this area. Just over 40 % of respondents also would have liked support to improve their confidence and self-esteem and deal with anxiety and depression and with finding a job after completing treatment.

Support needs identified through the interviews and focus groups with young people added detail to the needs recognised in the survey and included coping with the future, body image, fertility, and fatigue; fear of potential relapse, feeling unwell or experiencing worrying symptoms; and resumption of work or education, self-esteem and re-establishing friendships with healthy peers. The young people wanted reliable and trustworthy jargon-free cancer-related information with the opportunity to ask questions if they were unable to fully understand the information.

Emotional (e.g. anxiety and depression) and psychological (e.g. confidence and self-esteem) support needs also emerged from the discussions with health care professionals. Parents attending focus groups indicated they would also have liked support including how to cope and what to do when their teenage or young adult child was rejected or abandoned by their friends, information on treatment side effects and helping their child cope with their education.

### Desirability of online self-management

The survey results indicated that the majority of the sample had access to the internet and would be interested in accessing online self-management information in addition to other more personal support mechanisms. The majority (92 %) of the survey sample had access to the internet and regularly accessed Facebook. YouTube was the second most popular website (46 %), followed by Twitter (41 %) and Jimmyteens.tv (a platform that shares short films made by teenagers and young adults who have been affected by cancer) was accessed by just over 20 % of respondents.

Most survey respondents (84 %) wanted to access support information via safe internet websites. Whilst many would choose to speak directly to an individual for their support concerns, a large proportion of the young cancer survivors (75 %) who completed the online survey indicated they had no concerns about using a self-management website resource. The survey participants were asked about the devices they owned for accessing the internet. A laptop was the most commonly used device (by 88 %) followed by a smart phone (80 %). Most focus group respondents said they would use an app version of a self-management site if it was available

…a lot of us our age have smart phones and things, to be able to just sort of grab the information you need in a few minutes would be pretty good (TYA).

The wider expert group interviewed were supportive of a web-based self-management resource to meet information and support needs. However, parents and health professional respondents were keen to emphasise that any resource should complement, rather than replace, the important face-to-face consultations.

Health care professionals felt that a web-based self-management resource could enhance survivors’ feelings of control and responsibility by offering the potential for accessing information and support, and the capacity to self-manage at a time when they most needed it. Some professionals recognised the positive impact that a web-based self-management resource may have on their own clinical practice:

it would help me become more aware of the issues that are there for the kids, and that will help me when I actually see them (HSP).

Cancer survivors’ concerns in respect to online self-management were discussed in the focus groups. Similarly to the survey responses, the concerns were limited and were related to a preference for face-to-face contact, worries about privacy and confidentiality, and wanting to forget about having cancer after the treatment ended.

Yes, as like I said before, I don’t believe you’d get the same standard of emotional support as you would through face to face contact. (TYA)

They also highlighted the lack of emotional connection and the potential for gossip and hurtful comments:

*True emotion and feelings cannot be conveyed over the internet. (TYA)*

The potential to be overwhelmed with information was also highlighted:

*…it’s easy to be swamped with information that may not be relevant (TYA)*

Concerns raised by the health care professionals included the risk that survivors may become reliant on the online cancer discussion forums for support and social contact, which might delay them to moving forward with their lives and establishing and re-building other relationships. The reliability of information provided by teenage and young adults to each other was a concern for health care professionals, as well as issues of security and confidentially and the potential for online bullying.

### Potential features of an online system

**Fig. 1:**
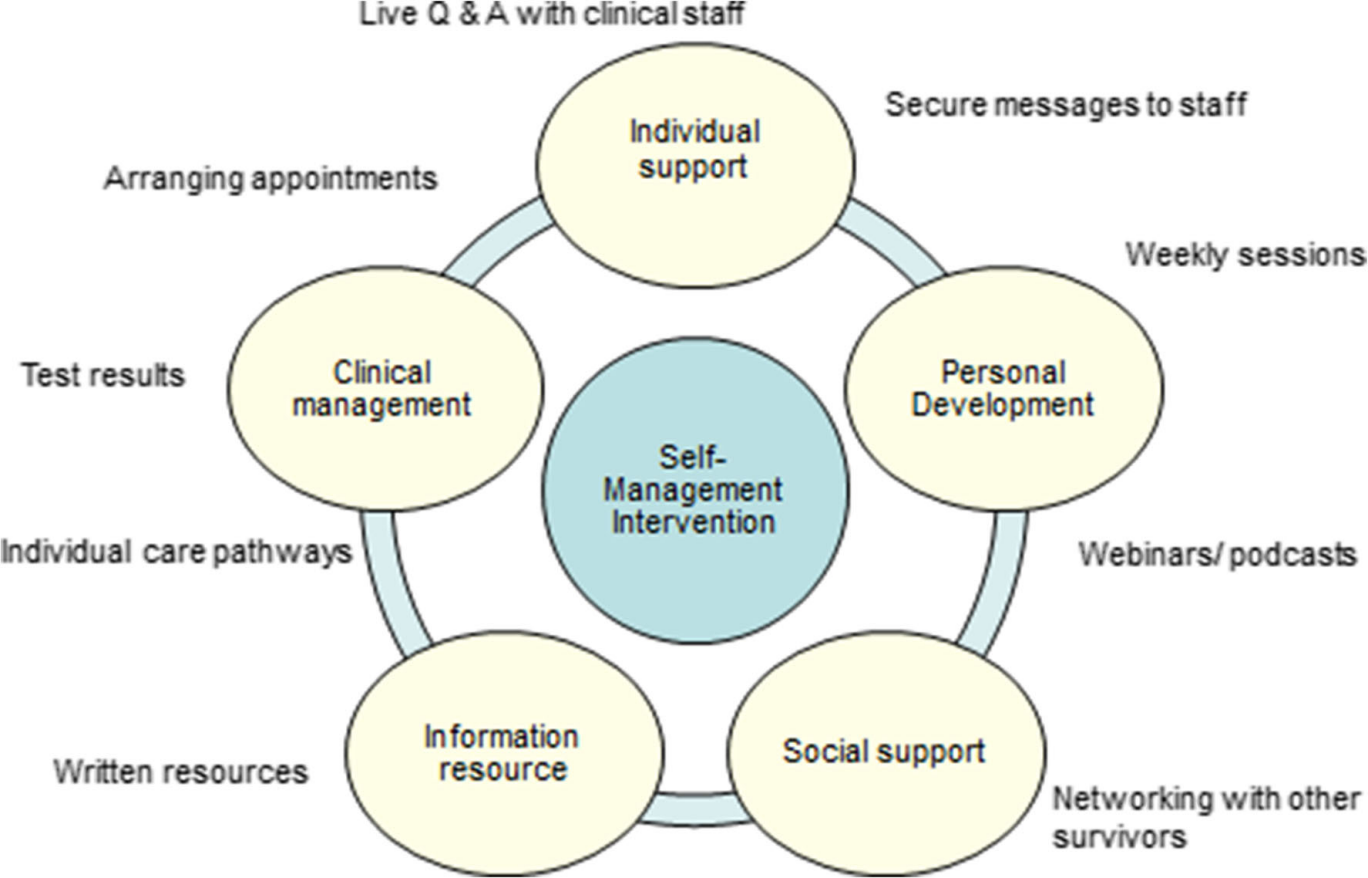
Potential online self-management model

The survey and interview participants were presented with a range of potential features that may be included within a self-management system (as illustrated in Fig. 1.) This included support functions, personal development features, clinical management functions and information content.

The young survivors indicated interest (through the survey, focus groups and interviews result) in clinical, information and social support features from a self-management system. The importance of the web resource being interactive and not solely focused on cancer experiences was emphasised by the survivors’ parents interviewed. Table 2 summarises the various self-management features against the views of the participant groups. The percentage of survey participants that would use each feature if it were available is highlighted, as well as a summary of each participant groups’ views on the desirability of that feature. The IT professionals commented on the technical feasibility.

**Table 2:** Potential features of an online self-management system and desirability to stakeholders

Self-management system features	Survivors survey (% that would use)	Survivor interview and FG findings	Parent interview findings	Health care professional interview findings	IT experts interview findings
Personal development					
Education/development	46 %	Recognised the need for support in a variety of areas; they would use an online system comprising of different features	Saw the benefit of online self-management and also wanted guidance on how to support their child	Considered useful to have a system at the end of treatment to provide support, and enhance self-esteem and confidence to self-manage	Straightforward to deliver through a range of delivery mechanisms Video podcasts are memory intensive
Case studies	52 %				
Webinars/podcasts	19 %				
Individual support					
Ask a doctor or nurse (private)	57 %	Considered a convenient way to access information and support without going to the hospital	Supported being able to ask questions of the clinical team online	Support is already provided by text message. Concerns about implementation and the ability to respond rapidly enough to survivor concerns	Technically possible by email through the Trust. Public discussion could provide governance issues
Live Q&A (public, e.g. Twitter)	62 %				
Clinical management					
Making appointments	76 %	Seen positively as a way to manage appointments and check appointment times	Regarded automated text message reminders about hospital appointments as useful	Seen as a good idea. Survivors can be reluctant to ring up and make appointments This would reduce the number of checks made about appointment times	Technically feasible, requires interfacing with other patient systems
Cancelling appointments	67 %				Straightforward to cancel, more complex to re-book
Appointment reminders by text	76 %				Appointment reminders about the time and location of appointment are straightforward
Looking up results	57 %	Generally not in favour as results need to be explained, not just provided	Only one parent was in favour of their child being able to access test results online	Not in favour. The need for clinical interpretation and emotional support was emphasised	Technically feasible. A clinician would need to review the results and then place in a secure area for access by the patient
Social support					
Patient–patient communication	52 %	Mixed views, reflecting personal perspectives on online communication and social networking	Generally supportive of social networking functions enabling their child to interact with other survivors	Peer to peer support is considered important	Governance issues around confidentiality, access, control and moderation of posts. Best hosted outside of the NHS

#### Education and development resource or intervention

The health care professionals interviewed stressed the need for credible information. The TYA felt that information on the internet was not tailored to young people, or contained distressing information such as mortality rates, or contained irrelevant information such as homoeopathic cures:

I think there’s almost too much on the internet isn’t there, and they’re all saying slightly different things, so it’s like what do I believe in and what’s the right thing (TYA).

Forty-six percent of survivors were interested in the provision of education and developmental material online. From the professionals’ point of view, an online self-management course was regarded as useful for some patients particularly in answering questions for those that have reached the end of their treatment.

Short-term weekly online courses followed by a break were suggested by health professionals to encourage commitment. It was identified that given the variability in the position of individuals on treatment pathways and potentially small numbers, there could be logistical problems with providing individualised weekly courses or a requirement to run the course at regular intervals to group patients together. It would also require resources to develop, deliver and moderate regular self-management course/sessions.

#### Appointment management

Many teenage and young adult cancer survivors would be willing to use an online self-management resource for managing appointments (76 %). The capability to look up, make or cancel appointments online was seen to be technically feasible, as was the provision of text/SMS or web-based appointment reminders. Health care professionals felt that online management of appointments could empower survivors and be a convenient mechanism for making follow-up appointments. They also noted that it may address issues with appointment non-attendance and reduce the time spent relaying appointment information. Challenges to implementation noted by IT professionals were interfacing the self-management system with the NHS Trust patient booking system and managing the complexity of several related appointment (e.g. a scan, a blood test and a meeting with special nurse).

#### Ask a doctor or nurse

The capacity to ask questions of a nurse or doctor either on a one to one basis or on a chat forum was discussed. Over 60 % of the survey respondents were interested in using live chat with a medical professional, and 57 % would like to be able to ask a doctor or nurse a question via secure messaging system. The health care professionals felt that the effectiveness of this type of support was dependent upon the young person’s trust in the clinician providing support. Logistically, it was recognised by the expert participants that a live chat or weekly time could be hard to manage if patients did not use it regularly, and also to schedule around patient educational or work commitments. Furthermore, clinicians may be reluctant to give advice with potentially incomplete case information. In terms of the security requirements of an NHS system, a secure email-based system (i.e. not live) would be more appropriate.

The desirability of working in this way was inconsistent amongst health care professionals, due to willingness to communicate with patients in this way, efficiency of the process and the time commitment and resources required. Concerns were raised regarding overuse by anxious patients, the potential to discourage patients from making direct contact with a health care professional and maintaining professional boundaries and standards.

#### Accessing test results

There were mixed views on the capacity to look up test results for routine issues. Fifty-seven percent of the survey respondents reported that they would use this feature if it were available. Young people in the FGs recognised the importance of results being explained and not just given. Only one parent was in favour of their child being able to access test results online. The clinical professionals interviewed were generally not in favour of test results being posted online because of lack of understanding of how to interpret them:

Results are not just about numbers, results are about interpretation of the significance of the numbers or the significance of a finding on a CT scan. If you find something that actually you don’t have that knowledge to be able to make that interpretation then that causes complete anxiety (HSP).

Health care professionals felt that accessing past results could be useful, both for the patient, who may not remember all of what has been said during a face to face appointment, and for support workers, who could access them with the patient and talk them through any patterns. This could be empowering for the patient as long as the results have already been explained. From a technical and information governance perspective, access to results would only be possible if a clinician has already reviewed the results and then posted them online in a secure area. It would not be appropriate for a patient to go directly into the results system, and there would be challenges to address in terms of communication between NHS systems.

#### Patient to patient communication

Around half of all survey respondents indicated that they would like to link up with other teenage and young adult cancer survivors in similar situations (52 %). Focus group respondents who had written personal blogs during their treatment found it therapeutic and felt it could help others find out what it like to experience cancer.

It’s just easier to like write everything out, like how I was feeling and stuff, and let everyone know at the same time (TYA).

The value of online social support from peers who had gone through similar cancer experiences was recognised across stakeholder groups. The health care professionals identified that social networking features could connect people from a wide geographical area and that providing some clinical features online could help reduce costly and time consuming travel to the hospital for routine appointments. They indicated that some young survivors are reluctant to return to hospital for emotional reasons, as they may experience distress as they remember their cancer diagnosis and treatment experience.

However, some participants indicated that they would prefer to meet up face-to-face before they would feel comfortable communicating online with other survivors. All focus group respondents and 81 % of survey participants indicated that they would use a closed/private group/forum to speak to others about cancer.

Health care professionals raised the issue that young people may frighten or undermine the confidence of others by talking about perceived poor care or “bad” doctors or nurses. The IT professionals interviewed highlighted the conflict between clinical, training and social functions of a self-management system, and the associated NHS information governance issues. Access and control issues would also need consideration in terms of patient to patient communication and risks associated with confidentiality. Many chat sites allow anonymity; however, a site with clinical functionality or training may not have this. Equally, there is a need to protect patient confidentiality. The IT professionals therefore advised that it was not appropriate to have social networking capacity within an NHS Trust site. If the self-management system was outside of the Trust, moderation and a code of conduct would still be required.

#### Additional features of an online self-management resource

Other potential features mentioned by the health care professional participants included a summary of treatment completed and an agreed follow-up plan to include not only medical issues but also rehabilitation: success stories, a diary of events and live interactive sessions, e.g. with a psychologist or coach. Being able to access an online treatment summary and follow-up plans was indicated as desirable features by the young survivors interviewed.

### Perceived risks and benefits of online self-management

The interviews revealed a number of perceived benefits and risks to features of an online self-management system. The benefits and risks perceived by the professionals interviewed are summarised in Table 3.

**Table 3:** Perceived benefits and risks of online self-management support

Perceived benefits	Perceived risks and challenges
Impact on the survivor	
• Enables networking and peer interaction amongst survivors	• Loss of contact with vulnerable people who will not ask for help and use the system
• Helps survivors feel like they are no longer a patient	• Poor clinic attendance and weaker survivor follow-up
• Provides access to resources 24 h a day	• Limited accessibility for young people with learning difficulties, brain tumours, memory problems, ADHD or who are vulnerable
• Reduced time before follow-up of individual issues	• Limited accessibility for young people without a smart phone or access to a PC
• Reduced re-admittance after discharge as problems tackled early, or without the need for admittance	• Online safety and confidentiality risks for survivors
	• Poor resourcing leads to lack of support and damages relationships with survivors
Logistics and delivery of care	
• One central, controlled and reliable resource	• Engaging and retaining young people
• Reduced waiting times as survivors visit the hospital less frequently	• Heavy clinical input required for developing and tailoring the content
• Faster access to support leading to early avoidance of problems	• The time required to moderate forums
• Improved geographical reach to dispersed groups that may not attend face to face events	• The complexity of providing
• The potential of the model to be transferred/used for other age groups and clinical areas, e.g. congenital heart defects, allergies, etc.	• A resource for small patient numbers at different parts of the treatment pathway
Financial and resourcing	
• Less resource hungry than face to face contact	• The financial outlay required for set-up
• Lower ongoing costs than face to face contact Providing hospitals with a means to innovate and increase technological usage	• Having to close down the system due to resource issues

The IT professionals saw no problem with the content and feasibility of the proposed self-management resource and potential features, noting that many of the elements already exist. It was highlighted that the appropriate means of hosting a self-management resource is complex in the UK, given governance constraints within the NHS and a requirement for N3^2^ compliance for certain functionality. As with all NHS Trusts, a key priority is patient confidentiality. Data protections laws and information governance procedures affect the passing of confidential information in or out of an NHS Trust. Systems managed and hosted by the NHS Trust have to automatically conform to current security and protection protocols.

A self-management intervention (in terms of a standard informational and developmental resource) is straightforward to develop and host. Therefore, both the IT and clinical participants advised separating systems, for example in terms of social networking and patient: patient interaction, from clinical support. This would be more straightforward to manage in terms of implementation and governance, but also ensure credibility of the information sources available.

## Discussion

Few UK studies have described the information and support needs of teenage and young adult cancer survivors. This paper has explored the support needs from a multi-stakeholder perspective and considered the potential for these needs to be met through a web-based self-management resource.

Teenager and young adult cancer survivors can feel isolated and abandoned at the end of treatment [19, 31]. This study has found that young survivors need ongoing emotional and psychological support as well as specific and relevant cancer-related information. The survivors involved in this study had worries about the future, potential relapse, body image, self-esteem, losing friends, fertility and fatigue. Many of these concerns were found in a recent qualitative systematic review and meta-synthesis involving 17 TYA cancer experience studies [32]. Many of these concerns have been reported by adult cancer survivors [31, 33–35], but there are also concerns specific to this age group to be addressed. A structured self-management course/resource to support the transition from treatment to survivorship could provide reliable materials to teenage and young adult cancer survivors in a number of formats, for example a weekly course, written materials, webinars or video podcasts.

End of treatment is a time when ongoing emotional and practical support is required. Support was shown from the health care professionals interviewed for a reliable and credible web-based resource that they could refer young people to. An online solution that is accessible at any time is likely to be beneficial for helping young cancer survivors take greater responsibility for self-managing their recovery, help them easily find reliable and trustworthy information in one place, and connect with other teenage and young adults with similar cancer experiences. Whilst many aspects of the support needed could be incorporated into a web-based self-management resource, the need for continued face to face direct interaction with support services was evident. Online self-management should therefore be regarded as a complementary, rather than alternative means of support with the potential to enhance the post-treatment experience whilst tapping into the technology preferences of young people.

The importance and benefit of young people having the opportunity to share feelings with others who are going through similar experiences emerged from the findings. Many of these benefits have been previously linked to social support whether it be through online support forums, e.g. TYA Facebook group, *Realshare* [15, 25]; face-to-face groups, e.g. four day retreat camp [13]; or activity groups [16]. The survivors involved in this study indicated a strong interest and limited reservations about online peer support via a web-based self-management resource. A *structured* approach to providing the peer support with the involvement of a trained moderator is likely to be the most effective model. Brunton and Panteli [15] found that when the online *Realshar*e site was moderated, the teenage and young adult cancer survivors gained more benefit and were more likely provide and receive emotional support.

As well as information and support, the young survivors were interested in online features that might facilitate their interactions with the health care system such as appointment systems and contact with a medical professionals. There was general support for a more comprehensive web-based self-management support system, with a range of developmental, support, clinical and social features, beyond those available through information-based sites and social networks. Access to medical professionals is desired by some young people, but would be challenging for clinical teams to manage. A scheduled and secure course-based environment may facilitate this. Further exploration is needed of the strong desire for online contact with health professionals. There is potentially a mismatch between young people’s experiences and expectations of technology and that of the health and social care professionals that support them. There may be an additional support required for professionals to use various modes on online communication appropriately.

Despite some interest in online access to test results from young people, the provision of test results was considered best provided face to face. A log of past results is more achievable and appropriate. Accessing medical records online is a cornerstone of the NHS Information Revolution. Further work is needed to explore this desire for online results and how this might be facilitated, and support provided to patients to enable appropriate interpretation without causing undue stress and anxiety.

Whilst information resources have been developed elsewhere, a comprehensive intervention in this area has not been evidenced by the other systems reviewed. A self-management intervention in terms of a standard informational and self-development resource should be straightforward to design and implement, in terms of the technology and surrounding governance requirements. Integrating further elements that might require access to personal information and interfacing with existing NHS Trust systems or social networking within Trust systems is more complex. It is recommended therefore that patient to patient interaction on a social basis is kept distinct from clinical functions as it avoids conflicts in terms of patient confidentiality and hosting requirements. This view is reflected by findings from the *Realshar*e online forum, where clinical and social aspects of the site were kept separate [15]. The continued use of Facebook for social contact moderated and supported by a code of conduct may be appropriate given young people’s existing engagement with the site. Self-management from a learning and development perspective could be successfully integrated with either social or clinical functions.

Ease of use and accessibility of a self-management resource on a range of platforms, particularly smart phones, is important when introducing and encouraging frequent access to an online space. Basing the format and look on sites that are familiar to young people and ensuring interactivity can help increase use and acceptance [36]. Ease of use and attractive presentation are keys to user acceptance; embedding both key visual elements and functionality within a system can help its uptake by the intended user group [37]. Web design elements should also take into account inclusivity, factoring in that the end users may have cancers that affect their ability to use technology, equally the suitability of the internet for vulnerable users should be considered.

It is clear that some form of web-based resource with a range of functionality would be valuable. Ongoing consideration is required of how it fits within existing services and how it enhances the quality of the service from the user and clinician perspectives. This approach to self-management will require resolution of governance and security issues for practical implementation.

The study described has several methodological limitations. The online survey sample was recruited online (via email, Facebook and text message); it is recognised, therefore, that this sample are biased towards technology and positive about communicating in this way. This study has considered a small and geographically local sample in the Southampton area. A larger sample drawn from a national pool of survivors would help clarify the experiences and perspectives of the broader TYA survivor population. Equally, it relied upon convenience sampling and a non-random sample of health care and IT professionals that were accessed through the UHS UK and their existing network. As an exploratory study, the findings offer a preliminary understanding of the context of online self-management for TYA survivors and begin to scope out the requirements for local system development. However, for broader application, the national picture and a wider range of participant/user views should be considered to exploit maximum benefit from existing network a self-management resource.

## Conclusions

There is a growing adoption of technology within NHS Trusts that supports patients in their own homes; this will increasingly include patient self-management. Through this research, it was intended to better understand the experiences, needs and wishes of teenage and young adult cancer survivors in terms of online support. The study has also highlighted some of the associated issues with self-management from the perspective of health and social care and information technology professionals.

## Footnotes

1 A UK charity supporting children, young people and their families in dealing with cancer. Their community workers provide practical and emotional support to young people, helping them cope with the emotional impact of cancer, social isolation and education, employment and training issues.

2 N3 = NHS national broadband network linking all NHS-based organisations allowing secure communication
